# Comparing Perspectives on Traditional and Complementary Medicine Use in Oncology: Insights from Healthcare Professionals and Oncology Patients in Western Mexico

**DOI:** 10.3390/curroncol32020071

**Published:** 2025-01-28

**Authors:** Gustavo A. Hernandez-Fuentes, Juan de D. Gómez-Bueno, Verónica M. Pérez-Santos, Imri J. Valle-Capitaine, Paula M. Villaseñor-Gonzalez, Cristopher J. Hernández-Zamorano, César G. Silva-Vázquez, Miriam de la Cruz-Ruiz, Janet Diaz-Martinez, Idalia Garza-Veloz, Iram P. Rodriguez-Sanchez, Margarita L. Martinez-Fierro, José Guzmán-Esquivel, Fabian Rojas-Larios, Ivan Delgado-Enciso

**Affiliations:** 1 Department of Molecular Medicine, School of Medicine, University of Colima, Colima 28040, Mexico; ghfuentes@ucol.mx (G.A.H.-F.); jgomez91@ucol.mx (J.d.D.G.-B.); vperez20@col.mx (V.M.P.-S.); ivalle@ucol.mx (I.J.V.-C.); pvillasenor1@ucol.mx (P.M.V.-G.); chernandez21@ucol.mx (C.J.H.-Z.); frojas@ucol.mx (F.R.-L.); 2 State Cancerology Institute of Colima, Health Services of the Mexican Social Security Institute for Welfare (IMSS-BIENESTAR), Colima 28085, Mexico; cesar.silva2359@alumnos.udg.mx; 3 Faculty of Chemical Sciences, University of Colima, Coquimatlan 28400, Mexico; 4 Oficina de Investigación en Salud, Servicios de Salud Del Instituto Mexicano del Seguro Social Para el Bienestar (IMSS-BIENESTAR), Colima 28085, Mexico; miriam.delacruz@imssbienestar.gob.mx; 5 Department of Dietetics & Nutrition, Robert Stempel College of Public Health, & Research Center in Minority Institutions, Florida International University, Miami, FL 33199, USA; jdimarti@fiu.edu; 6 Molecular Medicine Laboratory, Academic Unit of Human Medicine and Health Sciences, Autonomous University of Zacatecas, Zacatecas 98160, Mexico; idaliagv@uaz.edu.mx (I.G.-V.); margaritamf@uaz.edu.mx (M.L.M.-F.); 7 Molecular and Structural Physiology Laboratory, School of Biological Sciences, Autonomous University of Nuevo Leon, San Nicolas de los Garza 66455, Mexico; iram.rodriguezsa@uanl.edu.mx; 8 Clinical Epidemiology Research Unit, Mexican Institute of Social Security, Villa de Alvarez, Colima 28984, Mexico; jose.esquivel@imss.gob.mx; 9 Robert Stempel College of Public Health and Social Work, Florida International University, Miami, FL 33199, USA

**Keywords:** traditional medicine, alternative medicine, oncology patients, healthcare professionals, rural communities, cultural practices, complementary therapies

## Abstract

Traditional and complementary medicine (T&CM) plays a significant role in healthcare practices among healthcare professionals and oncology patients in Mexico, reflecting its cultural importance. This study aimed to analyze the prevalence, frequency, and factors associated with T&CM use in these two groups, highlighting the differences in practices and perceptions. A total of 382 individuals participated, including 152 healthcare professionals and 230 oncology patients. The findings revealed that while T&CM use was similarly prevalent among healthcare professionals (85.7%) and oncology patients (90.8%), frequent use (≥2 times per week) was significantly higher among patients (46.3%) compared to healthcare professionals (19.1%, *p* < 0.001). Healthcare professionals showed a preference for non-conventional nutritional interventions (32.5%) and yoga (14.6%) while oncology patients favored plant-based remedies (73.6%) and the consumption of exotic animals and venoms (4.8%). Females were more likely to use T&CM across both groups, with a stronger association among healthcare professionals (AdOR 3.695, 95% CI 1.8–7.4). Oncology patients were less likely to understand T&CM concepts and were more commonly associated with lower socioeconomic status and educational attainment. These findings underscore the importance of considering cultural and demographic factors when integrating T&CM into conventional medical care, especially in regions where T&CM remains widely practiced and trusted.

## 1. Introduction

Traditional medicine (TM), encompassing a wide range of cultural practices, therapies, and healing methods, continues to play a significant role in global healthcare systems. Defined by the World Health Organization (WHO) as follows: “It is the sum of the knowledge, skill, and practices based on the theories, beliefs, and experiences indigenous to different cultures, whether explicable or not, used in the maintenance of health as well as in the prevention, diagnosis, improvement or treatment of physical and mental illness” [1]. In the same sense, according to the WHO, complementary medicine (CM) or “alternative medicine” refers to a broad set of health care practices that are not part of that country’s own tradition or conventional medicine and are not fully integrated into the dominant healthcare system [1]. They are used interchangeably with traditional medicine in some countries. In this way, the terms are grouped into traditional and complementary medicine (T&CM) that includes herbal remedies, acupuncture, spiritual healing, and various holistic practices and the consumption of parts of exotic animals or secretions) [1,2,3,4]. Although modern medicine has become the dominant healthcare approach worldwide, T&CM remains relevant, particularly in rural areas and communities deeply connected to indigenous healing traditions [5]. In Mexico, T&CM is interwoven with the nation’s history and cultural identity, reflecting a blend of indigenous and colonial practices that continue to shape healthcare choices today [6,7,8].

The integration of T&CM into Mexico’s healthcare system has gained momentum in recent years, with many patients seeking alternative therapies alongside or in place of conventional treatments [9,10,11]. This reliance on T&CM is influenced by the country’s socioeconomic and geographic disparities. According to the National Institute of Statistics and Geography (INEGI), 56.6% of Mexico’s population belongs to the lower class, 42.2% to the middle class, and only 1.2% to the upper class [12]. These economic inequalities, combined with the limited availability of conventional healthcare in rural regions, contribute to the widespread use of T&CM. For example, in the state of Colima, 79% of the population lives in urban areas, and 21% resides in rural communities [12]. Rural populations, where healthcare infrastructure is often lacking, are more likely to engage with T&CM, preserving traditional knowledge and practices passed down through generations [12].

Colima’s economic landscape further underscores the relevance of T&CM. In the third quarter of 2022, the state’s total economic activity declined by 0.6%, ranking it second to last nationally. However, activities linked to agriculture, forestry, fishing, and hunting—sectors crucial for sourcing natural remedies—showed a 5.7% annual growth, positioning Colima ninth in the nation for these activities. This economic context highlights the practical and cultural significance of T&CM, as many remedies derive from locally sourced natural resources, maintaining its accessibility and relevance [12].

Globally, the perception of T&CM has evolved, with increasing recognition of its role in addressing healthcare gaps and improving quality of life (QoL) [13]. Research demonstrates that T&CM not only alleviates physical symptoms but also contributes to emotional and psychological well-being, particularly for patients managing chronic illnesses such as cancer [14,15]. In oncology care, QoL assessments have gained prominence, emphasizing the importance of holistic strategies, including T&CM, to meet patients’ complex needs [16,17]. In addition to the positive effects of using T&CM, it is critical to address the negative side of using T&CM in patients with cancer, particularly drug–herbal interactions, and the adverse effects of using TM in oncology patients with ongoing treatments [5,9,18].

Despite its cultural and therapeutic value, perceptions of T&CM vary widely, particularly between healthcare professionals and patients. Healthcare providers, such as physicians and nurses, often approach T&CM with skepticism due to its perceived lack of scientific evidence [19,20,21]. Rooted in evidence-based medicine, these professionals may view traditional therapies as outdated or unproven, although some acknowledge their cultural significance and potential to complement biomedical treatments like various reports of these effects [22,23,24]. Conversely, oncology patients often hold more favorable views of T&CM. Many seek complementary and alternative therapies alongside conventional cancer treatments, aiming to alleviate symptoms, manage side effects, or find emotional and spiritual support. Patients, especially those with advanced cancer, frequently turn to T&CM as a way to regain a sense of control over their health and enhance their overall QoL. Studies suggest that patients value T&CM not only for its physical benefits but also for the emotional comfort, spiritual connection, and empowerment it offers during their healthcare journey [25]. This divergence in perceptions—between healthcare professionals prioritizing scientific validation and patients seeking holistic support—reflects a broader interplay of cultural beliefs, healthcare access, and individual experiences [16]. In Mexico, and particularly in Colima, understanding these differing viewpoints is crucial to fostering integrative medicine care models that respect cultural practices while ensuring safety and efficacy in treatment. More accurately integrative medicine is an approach to medical care that recognizes the benefit of combining conventional (standard) therapies (such as drugs and surgery) with complementary therapies (like acupuncture and yoga) that have been shown to be safe and effective [26].

The objective of this study is to compare the perspectives of healthcare professionals and oncology patients regarding the use of traditional and complementary medicine (T&CM) in cancer care. By examining perceptions of T&CM’s efficacy, safety, and relevance, this research aims to illuminate the cultural, socioeconomic, and healthcare factors influencing its use. Furthermore, it seeks to explore how T&CM shapes the patient experience in oncology settings, particularly in the context of Colima, Mexico, where traditional practices remain deeply rooted in the region’s history and culture. The study also aims to assess the feasibility of adopting T&CM within the current healthcare system, considering the existing limitations and the prevailing medical ideologies in a future trend to an integrative medicine care model.

## 2. Materials and Methods

### 2.1. Study Design and Population

This cross-sectional survey and observational study was conducted to investigate the use of traditional (TM) and complementary medicine (CM) among oncology patients and healthcare professionals in a tertiary care oncology hospital located in Western Mexico. The hospital (State Cancerology Institute of Colima, Mexico, IMSS-BIENESTAR), serving the regions of Colima, Jalisco, and Michoacán, provides a diverse patient demographic, making it an ideal setting for assessing T&CM usage patterns and associated factors. This study has been ethically approved by the Research Ethics Committee of the State Cancer Institute of Colima (approval number CEICANCL14072023-USCPCCAN-12, 14 July 2023). Each participant was asked to give their written informed consent, making it clear that their participation was voluntary and anonymous. The study was reported according to the Strengthening the Reporting of Observational Studies in Epidemiology (STROBE) Statement guidelines for reporting observational studies [27].

### 2.2. Variables and Measurement

The dependent variable in our study was the use of traditional or complementary medicine (T&CM), which refers to a wide range of health practices that are not integrated into the conventional healthcare system. These practices may be rooted in regional culture or tradition (traditional medicine) or may be considered non-traditional (complementary or alternative medicine). In order to clarify the terminology used and also contextualize how these concepts were operationalized in our study and analyses, the following terms were considered. Herbal Medicine: A type of medicine that uses roots, stems, leaves, flowers, or seeds of plants to improve health, prevent disease, and treat illness [26]. Plant-Based Remedies: Preparations derived from plants, often categorized under TM or CM depending on the context and use [1]. Non-Conventional Nutritional Interventions: Refers to dietary practices, supplements, or regimens that lie outside conventional dietary guidelines, typically associated with CM [28]. Consumption of exotic animals/venoms: A branch of traditional medicine in many cultures, especially in indigenous and rural communities, involving the use of animal derivatives such as fats, bones, skins, venoms, and other components for therapeutic purposes [29]. Conventional Medicine: A system in which medical doctors and other healthcare professionals (such as nurses, pharmacists, and therapists) treat symptoms and diseases using drugs, radiation, or surgery. Also referred to as allopathic medicine, biomedicine, mainstream medicine, orthodox medicine, or Western medicine [26]. The definitions employed for complementary or alternative medicine are shown in Supplementary Material Section S1 [30,31,32,33,34,35,36,37,38,39,40,41].

The independent variable was the categorization of participants, either as oncology patients or as healthcare professionals working at the hospital where the patients receive treatment. All participants were asked in a face-to-face survey, with interviews conducted across all shifts at the medical center, including both patients and healthcare personnel. However, participant selection was not randomized, asking in addition to universal variables (gender, age, sociocultural aspects, and personal pathological history), if they consider that they know a definition of traditional and alternative medicine. After that, offering a definition of traditional and alternative medicine, they were asked their opinion on the subject, offering 5 options: (1) It works well for some diseases, but should be used together with conventional medicine. (2) It works well for some diseases, where it can be the only treatment. (3) It works well for all diseases and can be the only treatment. (4) I respect its use, but I would not use it. (5) I do not agree. We considered options 1, 2, and 3 as responses with positive opinions about T&CM. They are also asked about the use of this type of therapy at some point in their life and currently, delving into the types of therapies, products administered, frequency, and current form of consumption. Non-conventional nutritional interventions (e.g., elimination of fats, sugars, etc.) were considered as part of complementary medicine, according to previous reports [28]. The socioeconomic level was categorized based on the 2022 guidelines of the Mexican Association of Market Intelligence and Opinion Agencies (AMAI) [39,40]. Households were classified into three groups: A/B (high); C (middle level); and D/E (Low) [42].

### 2.3. Questionnaire Development

In the absence of a validated questionnaire aligned with our objectives, we developed a new instrument tailored to the needs of this study. The questionnaire was designed using a structured approach, incorporating both closed and open-ended questions to ensure comprehensive data collection while maintaining clarity and reliability. It consisted of 31 questions, divided into four key sections, each addressing a specific theoretical dimension identified through a detailed literature review and refined through expert validation.

The first section focused on universal, sociodemographic, and clinical characteristics to create a profile of the participants. The second section assessed participants’ knowledge of traditional and complementary (alternative) medicine, while the third explored their use of traditional and complementary treatments. The final section captured opinions and attitudes toward traditional and CM. The complete version of the questionnaire is provided in the Supplementary Material, and the validation process was carried out in Spanish.

To ensure the questionnaire’s content was relevant and clear, it underwent validation by a panel of experts, including two oncologists, four general practitioners, three psychologists, and three social workers. Each expert evaluated the questions for their relevance and clarity, and the Content Validity Index (CVI) was calculated for each section [43]. Questions with a CVI below 0.8 were revised or excluded to maintain the instrument’s quality and precision.

A pilot test involving 30 participants—10 healthcare professionals and 20 oncology patients—was conducted to identify and address any ambiguities in the questions. Feedback from this pilot test led to adjustments in the format and language of several items to ensure they were understandable to all participants. Data collected during the pilot test were not included in the final analysis presented in this study.

The theoretical dimensions of the questionnaire were further reviewed and confirmed by the expert panel. Based on these reviews, the questions were refined to ensure they accurately measured the intended constructs. The internal consistency of the instrument was assessed using Cronbach’s alpha, demonstrating its reliability for the study.

This meticulous process resulted in a well-designed and validated questionnaire capable of capturing the knowledge, usage patterns, and perceptions of traditional and alternative medicine among participants. The questionnaire is shown in Supplementary Information Section S2.

### 2.4. Selection Criteria

Inclusion criteria for oncology patients were to be aged 18–80 years with a confirmed cancer diagnosis regardless of clinical stage or therapy received, and attending the State Cancerology Institute of Colima. A consecutive sampling method was implemented, enrolling all eligible patients who met the inclusion criteria for their follow-up visits to the State Cancerology Institute of Colima for follow-up appointments to reduce selection bias. Healthcare professionals were recruited through a voluntary participation process. After being informed about the study’s objectives and procedures, they were invited to participate if they met the inclusion criteria. This group included physicians, nurses, and other paramedical and administrative staff who care for oncology patients and who work at the State Institute of Cancerology of Colima during the recruitment period. Recruitment took place between 2023 and 2024, ensuring a consistent and representative sample of the target population. Eligibility was determined through preliminary interviews and clinical evaluations prior to enrollment. Participation was strictly voluntary, with all individuals providing informed consent in compliance with ethical research standards. Exclusion criteria for both groups included unwillingness to participate, cognitive impairments, current hospitalization, current use of antidepressants, or diagnosed psychiatric disorders.

### 2.5. Sample Size Calculation

The sample size was calculated to detect a difference in frequent T&CM usage between oncology patients and healthcare professionals with a power of 80% and a significance level of 0.05. The sample size calculation was based on previous reports showing that 46.0% of oncology patients [24] and 29.6% of healthcare professionals [25] use a type of T&CM, which is the most widely used type of T&CM in Latin America, particularly in Mexico [19]. Based on these findings, the minimum required sample size was determined to be 136 participants per group [44,45]. The final sample of 382 individuals exceeded this requirement. A post hoc statistical power analysis was performed after the study was completed, showing that the consumption of herbal medicines was different between oncology patients and health personnel, resulting in a statistical power of 99.8% for “having ever used it” and 100% for “current use” (α = 0.05).

### 2.6. Statistical Analysis

After data cleaning, participants with incomplete responses or open-ended answers that were incomprehensible were excluded from the dataset. Subsequently, the normality of the data was evaluated using the Kolmogorov–Smirnov test. Continuous variables were summarized as means ± standard deviations (SD) and compared between groups using the independent Student’s *t*-test. Categorical variables were expressed as frequencies and percentages and analyzed using the chi-square test or Fisher’s exact test, as appropriate. Logistic regression models were used to identify factors associated with TAM usage. Adjusted odds ratios (AdORs) with 95% confidence intervals (CIs) were calculated. The backward stepwise method was employed to identify the most parsimonious model, with entry and removal probabilities set at 0.10 and 0.15, respectively. Separate models were created for all study participants, for healthcare professionals, and for oncology patients, to explore subgroup-specific associations. An SPSS Statistics version 20 software program was used for data analysis (IBM Corp., Armonk, NY, USA) [46]. ClinCalc version 1 was used to calculate sample size and statistical power [47,48,49] (https://clincalc.com/stats/Power.aspx; accessed on 2 December 2024). A *p* < 0.05 was considered statistically significant [49].

### 2.7. Limitations

Data on TAM usage were self-reported, which may be subject to recall or social desirability bias. Additionally, the cross-sectional design precludes causal inferences. Efforts were made to minimize bias by standardizing survey administration and corroborating clinical data where applicable [50,51].

## 3. Results

Regarding the validation of the instrument, it was concluded that the selected questions were clear, relevant, and adequately represented the dimensions of traditional and alternative medicine that the study aimed to evaluate. The reliability analysis demonstrated high consistency in responses across the different sections of the questionnaire. The overall Content Validity Index (CVI) was 0.91, reflecting excellent content validity. Additionally, the theoretical dimensions of the questionnaire were confirmed by the expert panel. The Cronbach’s alpha coefficient reached a value of 0.85, indicating strong internal consistency of the instrument. These results confirm that the questionnaire is both reliable and appropriate for measuring the objectives of the study. During the data cleaning process, 11 participants were excluded, leaving a final sample of 382 individuals for analysis.

Table 1 summarizes the general characteristics and the use of traditional and alternative medicine among oncology patients and healthcare personnel included in the study. Additionally, Table 2 provides a detailed breakdown of the types of cancer diagnoses and the conventional cancer care treatments received by the oncology patients. The cancer diagnoses among patients were as follows: breast cancer (46.1%, *n* = 106), cervical cancer (11.3%, *n* = 26), prostate cancer (11.3%, *n* = 26), colorectal cancer (8.7%, *n* = 20), ovarian cancer (6.1%, *n* = 14), non-Hodgkin lymphoma (2.6%, *n* = 6), thyroid cancer (2.6%, *n* = 6), lung cancer (2.2%, *n* = 5), and other types (9.1%, *n* = 21) (Table 2).

Table 1 shows that healthcare professionals were younger (mean age 42.3 ± 9.6 years) compared to oncology patients (mean age 56.0 ± 14.0 years, *p* < 0.001). Healthcare professionals also had a lower BMI (27.4 ± 4.6) compared to oncology patients (29.5 ± 5.5, *p* < 0.001). Overall, 87.7% (*n* = 335) of the participants reported having used traditional or alternative medicine at least once in their lifetime, with no significant differences between healthcare professionals (85.7%, *n* = 130) and oncology patients (90.8%, *n* = 209, *p* = 0.154). However, current and frequent use (defined as 2 or more times per week) was significantly more common among oncology patients (46.3%, *n* = 106) compared to physicians (6.5%, *n* = 10), nurses (33.3%, *n* = 24), and other hospital staff (15.4%, *n* = 12, *p* < 0.001, multigroup analysis).

Having a clear understanding or concept of what traditional or alternative medicine entails was significantly less common among oncology patients (39.6%, *n* = 91) compared to healthcare professionals (60.4%, *n* = 92, *p* < 0.001). In general, oncology patients were more likely to have other chronic diseases, lower educational attainment, and lower socioeconomic status (*p* < 0.05 for all comparisons). There were no significant differences between groups in terms of living with a partner or identifying as Catholic (*p* > 0.05 for both comparisons; see Table 1).

Regarding the use of conventional medicine among healthcare personnel, 22.8% reported using antihypertensive drugs, 10.6% used analgesics/anti-inflammatory drugs, 9.1% used antidiabetic medications, 9.1% used medications for hormonal disorders, 4.6% reported using immunotherapy, 3.0% used antilipemic drugs, and antidepressants/anxiolytics were used at the same frequency. Among cancer patients, chemotherapy was the most commonly used treatment (see Table 1).

### 3.1. Use of Traditional and Alternative Medicine

Although no significant differences were found in the overall use of alternative medicine between healthcare professionals and oncology patients (85.7%, *n* = 130, vs. 90.8%, *n* = 209, *p* = 0.154), Table 3 highlights notable variations in the types of therapies used by each group. The use of plants was significantly more common among oncology patients than healthcare professionals. Conversely, non-conventional nutritional interventions and yoga were more frequently practiced by healthcare personnel.

The use of animal products, parts, or even animal-derived substances like venoms for medicinal purposes is a notable therapeutic practice, especially in certain Indigenous and rural communities, and includes a variety of treatments that involve animal-derived products such as fats, bones, skins, and even animal parts like venom or flesh. The inclusion of this therapy is relevant, as it is still a common practice among oncology patients in some regions. The survey findings revealed that many patients reported using or consuming animal products, such as snake meat or stews made from animals like vulture, armadillo, or skunk, in an attempt to treat their cancer. These practices, which are often passed down through generations or learned from traditional healers, continue to be significant in the lives of some patients.

Interestingly, healthcare professionals also demonstrated awareness of these practices, and some even acknowledged having used or been informed about such therapies. These findings show a blend of traditional and conventional medicine in the healthcare context, highlighting the cultural relevance and the persistence of traditional treatments among oncology patients. The practice of consumption of exotic animals/venoms is important to explore because it represents an alternative therapeutic avenue that may complement, but also potentially interfere with, conventional cancer treatments.

Similarly, acupuncture and yoga are other traditional therapies that show varied use across the two groups. Acupuncture, which involves inserting thin needles into specific points of the body to alleviate pain and promote healing, is often reported as effective for managing symptoms like pain or nausea, particularly in oncology patients. Although its use was not significantly different between the two groups in this study, it remains a widely recognized alternative therapy. On the other hand, yoga, which combines physical postures, breathing exercises, and meditation, is more common among healthcare personnel, possibly due to their greater exposure to mindfulness practices for stress management and physical well-being.

### 3.2. Females Are More Associated with Traditional Medicine Use

A multivariate analysis shows that female gender is a factor associated with the use of traditional medicine (AdOR 2.8, 95% CI 1.4–5.3), as well as knowing the concept of traditional and alternative medicine (1.6, 95% CI 1.004–2.8). When analyzing the groups of oncology patients and healthcare professionals separately, female gender remained the only factor associated with the use of this medicine (see Table 4).

While Table 4 shows that the female gender is consistently associated with the use of traditional medicine in both oncology patients and healthcare personnel, other factors such as knowledge of traditional medicine did not show a strong association in the stratified analysis. These findings show that being female is a key demographic factor influencing the use of alternative treatments. Notably, the odds ratios are higher for healthcare professionals (3.695) compared to oncology patients (2.587), which could indicate a greater familiarity or acceptance of traditional medicine within the healthcare profession. This trend warrants further investigation into the reasons behind these differences, including cultural and educational influences.

### 3.3. Current and Frequent Use of Traditional and Alternative Medicine

**Figure 1:**
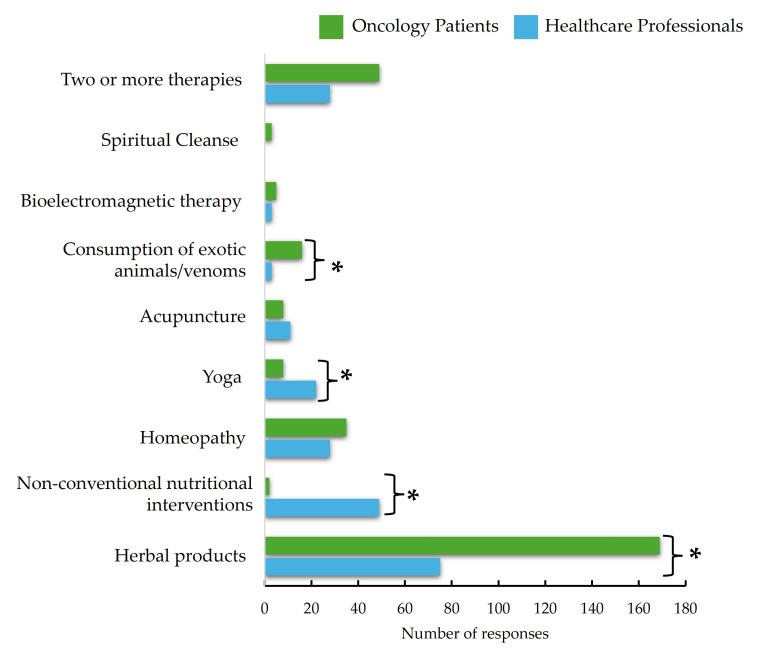
Comparison in Terms of Number of Responses Used Traditional and Complementary Medicine Types Between Healthcare Professionals and Oncology Patients. * Statistical difference at alfa = 0.05.

The frequent use of alternative medicine was significantly higher among oncology patients compared to healthcare professionals (46.3% vs. 19.1%, *p* < 0.001) (Table 5 and Figure 1). Specifically, the use of plants was notably more common among oncology patients (43.4%) than among healthcare professionals (15.8%) (*p* < 0.001). This pattern was also observed for homeopathy, with 9.2% of oncology patients reporting its use versus 3.3% of healthcare professionals (*p* = 0.036). Furthermore, the consumption of exotic animals and venoms was more prevalent among oncology patients (4.8%) than healthcare professionals (0.0%) (*p* = 0.004). Ayurveda, traditional Chinese medicine, zootherapy (therapeutic approach that involves interaction with animals), and osteopathy were not mentioned in any case, so it can be inferred that they are rarely used or not identified in the region.

Interestingly, non-conventional nutritional interventions were more frequently used by healthcare professionals (3.3%) compared to oncology patients (0.4%) (*p* = 0.039). These findings show that healthcare workers may be more inclined to explore alternative dietary practices or supplements. Additionally, the use of two or more types of alternative therapies was more common among oncology patients (13.2%) compared to healthcare professionals (6.6%) (*p* = 0.042). The results show that oncology patients are more likely to incorporate a combination of alternative treatments into their care regimen, possibly due to the comprehensive nature of their treatment and the desire for additional supportive therapies.

### 3.4. Multivariate Logistic Regression to Detect Factors Associated with Current and Frequent Use of Alternative Medicine

A multivariate analysis revealed that being an oncology patient increased the likelihood of using alternative treatments by more than six times compared to healthcare professionals (Adjusted Odds Ratio [AdOR] 6.5, 95% CI: 3.7–11.5) (see Table 6). Additionally, having knowledge or a concept about alternative medicine was also associated with its use (AdOR 2.6, 95% CI: 1.6–4.4). Conversely, being obese was a factor that reduced the likelihood of using alternative medicine (AdOR 0.6, 95% CI: 0.37–0.99).

When analyzing the groups separately, among healthcare personnel, having hypertension and being 50 years or older were factors associated with the use of alternative medicine. On the other hand, living with a partner and being obese were inversely associated with its use (see Table 6). Among oncology patients, having knowledge or a concept about traditional and alternative medicine increased the likelihood of its use by 2.6 times (AdOR 2.6, 95% CI: 1.5–4.5).

### 3.5. Opinion on the Use of Traditional and Complementary Medicine

Although only 60.4% of healthcare professionals and 39.6% of oncology patients reported being familiar with and able to define traditional and complementary therapies, all participants had an opinion on their use. The majority of respondents (64.9%) believed that “It works well for some diseases, but should be used alongside conventional medicine”, with this response being more prevalent among oncology patients (75.7%) compared to healthcare professionals (48.7%) (*p* < 0.001). The response “It works well for some diseases and can be a sole treatment” was selected by 24.3% of healthcare professionals and 7.8% of oncology patients (*p* < 0.001). Meanwhile, 9.2% of healthcare professionals and 3.0% of oncology patients believed that “It works well for all diseases and can be the only treatment” (*p* = 0.012). These responses were considered positive opinions toward traditional and complementary medicine, resulting in 82.2% of healthcare professionals and 86.5% of oncology patients expressing such views (*p* = 0.308) (see Table 7).

### 3.6. What and How People Consume Traditional and Complementary Medicine

A total of 73 products used in traditional or complementary medicine were identified, with 47 products reported by oncology patients and 37 by healthcare personnel. While a small proportion of individuals in both groups could not recall the names of the plants or products they consumed (4.1% of oncology patients and 7.7% of healthcare personnel), notable differences were observed in the types and frequency of use between the two groups (see Table 8).

Oncology patients predominantly consumed guanabana (*Annona muricata*), with 33.7% reporting its use. This aligns with its widespread reputation for potential anticancer properties in traditional medicine. Interestingly, no healthcare professionals reported using guanabana, suggesting a possible disconnect between lay practices and professional knowledge or preferences. Other popular products among oncology patients included maguey (*Agave* spp.) at 9.3%, *Aloe vera* at 4.1%, and *Kalanchoe pinnata* at 3.5%. The preference for these products may stem from their purported health benefits, including anti-inflammatory, immunomodulatory, and wound-healing properties.

Additionally, less commonly consumed items such as snake meat (2.9%) and turmeric (*Curcuma longa*, 1.7%) reflect the diversity of traditional remedies explored by this group, often in the hope of finding complementary therapies for their condition. The unique inclusion of animal-derived products, such as snake and iguana meat, highlights cultural influences in selecting remedies.

Healthcare professionals demonstrated a distinct pattern of consumption, with skunk vine (*Paullinia pinnata*, 9.3%) being the most frequently reported product. This plant is often associated with traditional medicinal uses such as treating inflammation or respiratory conditions. Other widely consumed products include chamomile (*Chamaemelum nobile*, 6.5%), cinnamon (*Cinnamomum verum*, 4.7%), and peppermint (*Mentha spicata*, 4.7%), all of which are commonly used for their soothing, digestive, or aromatic properties.

Unlike oncology patients, healthcare professionals showed a broader use of medicinal herbs that are typically integrated into everyday health practices rather than being associated with specific therapeutic claims for chronic or severe conditions. Notable examples include arnica (*Arnica montana*, 2.8%), ginger (*Zingiber officinale*, 2.8%), and noni (*Morinda citrifolia*, 2.8%).

The differences in consumption patterns may reflect varying motivations and accessibility. Oncology patients often turn to remedies believed to have curative or supportive effects against cancer, whereas healthcare professionals may use traditional products for general wellness or common ailments. Moreover, healthcare personnel’s broader range of herbal and plant-based remedies shows a greater familiarity with or with access to these products.

Interestingly, common plants like guava (*Psidium guajava*) and *Aloe vera* were reported in both groups, reflecting their universal recognition and accessibility. However, unique mentions such as turmeric in oncology patients and parota (*Enterolobium cyclocarpum*, 1.9%) in healthcare professionals highlight cultural and possibly professional influences in selecting traditional therapies.

The discrepancy between groups, particularly the absence of guanabana consumption among healthcare professionals despite its high use among oncology patients, raises questions about perceived efficacy and awareness. This highlights the importance of bridging gaps between patient preferences and healthcare recommendations to foster evidence-based integration of complementary therapies.

The diversity and motivations for using traditional and complementary medicine underscore the need for further research into their safety, efficacy, and cultural significance to support informed decisions in both clinical and personal contexts.

### 3.7. Women Use Traditional and Alternative Medicine More Frequently, Except in the Context of Cancer

When considering all subjects included in the study, women were found to have used traditional or alternative medicine at a higher rate than men (90.8% vs. 78.8%, *p* = 0.002). Women also reported current and frequent use more often (38.2% vs. 27.6%, *p* = 0.037). This trend persists among healthcare personnel, with 94.5% of women and 81.4% of men having used such therapies at least once (*p* = 0.017), and 22.9% of women versus 9.3% of men reporting current and frequent use (*p* = 0.040).

In oncology patients, the use of these therapies at least once was still more common among women (88.5%) than men (76.8%) (*p* = 0.029). However, current and frequent use as a complement to cancer therapy showed no significant difference between women (47.7%) and men (41.8%) (*p* = 0.272). This aligns with the results of the multivariate analysis, which revealed that while women are generally more likely to use these therapies at some point in their lives, other factors become more influential. Gender does not significantly impact the current and frequent use of traditional medicine when analyzed alongside other factors, particularly in oncology patients.

## 4. Discussion

This study highlights the widespread use of traditional and complementary medicine (T&CM) among oncology patients and healthcare professionals at an oncology hospital in Western Mexico, revealing significant differences in the types of therapies used and their frequency. The overall use of T&CM was high in both groups, but the patterns and preferences reflect contrasting cultural, educational, and professional influences. These findings contribute to a growing body of literature examining the role of T&CM in healthcare, particularly among populations managing chronic or severe illnesses [52,53,54]. Understanding the contrasting perspectives of patients and healthcare professionals is crucial for fostering communication and collaboration in oncology care. These differences reflect broader cultural, socioeconomic, and healthcare dynamics that influence the acceptance and integration of T&CM in cancer care. By identifying areas of alignment and tension, this research provides insights into the challenges and opportunities of integrating T&CM into mainstream healthcare practices, ensuring that both cultural preferences and scientific evidence are respected. In this study, nurses and other healthcare professionals were included due to their critical role in patient care. Nurses have direct, continuous interactions with patients, providing unique insights into patient needs and experiences [55]. They play a pivotal role in administering care, educating patients, advocating for vulnerable individuals, and coordinating treatment [55,56,57]. Given the study’s aim to assess the potential for an integrative medicine model, it is essential to involve all healthcare professionals [57]. Including nurses and other staff ensures a more comprehensive understanding of current practices and perceptions, facilitating the effective implementation of such a model.

The principal results of the study are in regard to the frequent use of herbal products among oncology patients, which aligns with prior studies emphasizing the cultural significance of plant-based remedies in Latin America [21,53,58,59]. Research conducted in Lisbon, for instance, reported that over 70% of oncology patients used herbal therapies during cancer treatment [44]. This is in contrast to findings from Asia and Europe, where herbal therapy usage among oncology patients is often lower, likely due to differences in cultural beliefs and access to other integrative therapies [60,61]. The high use of herbal products in oncology patients in our study may be driven by a desire for holistic or natural remedies to manage symptoms or side effects of cancer treatments [62,63].

Interestingly, healthcare professionals reported significantly higher use of non-conventional nutritional interventions, a pattern that might reflect their access to up-to-date dietary research and a more health-conscious behavior. This is in stark contrast to oncology patients, who showed minimal use of non-conventional nutritional strategies. This disparity may suggest a lack of awareness or accessibility to evidence-based nutritional interventions among patients, an area that could benefit from further patient education and intervention [62,64].

The consumption of exotic animals/venoms, though less common, presents an intriguing aspect of T&CM among oncology patients. Practices such as consuming snake meat or other animal-based stews underscore the persistence of deeply rooted indigenous and rural traditions. Consumption of exotic animals/venoms, though relatively rare, is a practice also reported in other regions, including Africa and Asia, where animal products are considered integral to traditional healing systems [29,65]. The findings imply that the consumption of exotic animals/venoms, although culturally specific, may have a significant role in cancer treatment. However, its potential interactions with conventional treatments should be further explored to ensure patient safety.

Both acupuncture and yoga were used less frequently, but there were interesting differences between the two groups. Healthcare personnel’s higher engagement with yoga could reflect their familiarity with the evidence supporting its benefits for stress reduction and mental well-being [66,67]. On the other hand, the moderate use of acupuncture among both oncology patients and healthcare professionals indicates a shared belief in its efficacy for managing symptoms like pain and nausea. Systematic reviews support the benefits of acupuncture in oncology care [68], though its adoption in low-resource settings like Mexico could be limited by accessibility and costs [68,69].

The study also examined gender as a factor influencing the use of T&CM. While a general trend suggests that women favor traditional and alternative therapies due to greater health-seeking behavior and cultural factors that encourage exploring holistic approaches, this trend diverged among oncology patients. No significant gender difference was observed in TAM use among oncology patients (89.3% in men vs. 91.2% in women, *p* = 0.762). This parity might be attributed to the shared urgency of managing cancer symptoms and the search for complementary treatments, which seem to override gender distinctions [70].

However, it is important to acknowledge the historical context of women’s roles in healing. Throughout history, women have often been central figures in traditional medicine, particularly in rural settings. In the Early Modern era, for example, women who were healers, or “wise-women”, were pivotal in village medicine, using herbs, poultices, prayers, and ointments to cure various illnesses [70]. This healing role, however, was threatened during the Renaissance when the medical profession sought to distance itself from popular culture and solidify its position as a specialized, regulated field. As the medical profession became more institutionalized, the work of female healers was increasingly marginalized. Women’s involvement in healing was not only restricted by the professionalization of medicine but also by accusations of witchcraft. Wise-women, who often acted as midwives and healers, were sometimes accused of witchcraft, a social mechanism used to push women out of medicine and discredit their roles in healthcare. This process reflects a broader historical trend of undermining women’s traditional roles as healers, reinforcing gendered power dynamics in the medical field [70].

In light of this history, the parity in T&CM use between male and female oncology patients in our study could suggest that despite the historical challenges women faced in the medical realm, modern healthcare contexts—especially those involving chronic or severe illnesses like cancer—highlight the value of holistic, integrative approaches. For many women and men alike, seeking complementary treatments is less about gender and more about the shared need for symptom relief and improving quality of life. Yet, it also underscores the broader cultural significance of women’s healing traditions, which have persisted despite historical attempts to marginalize them. This dynamic may help explain why both genders turn to T&CM in similar ways when faced with serious health challenges like cancer. In contrast, among the general population and healthcare personnel, women reported a higher adoption of T&CM [71]. This could be due to greater healthcare engagement, cultural norms, and perceptions of T&CM aligning with women’s health needs. The gender-based analysis suggests that interventions promoting informed use of T&CM should be tailored to gender-specific trends. For non-cancer populations, targeting women may yield higher engagement, while for oncology patients, strategies should focus on shared priorities like symptom management and quality of life [71,72].

The multivariate analysis revealed that female gender was the most consistent factor associated with TAM use across both groups. Adjusted odds ratios (AdORs) confirmed that being female plays a central role in influencing attitudes toward and use of traditional medicine: All participants: AdOR 2.816 (95% CI: 1.487–5.331, *p* = 0.001). Healthcare personnel: AdOR 3.695 (95% CI: 1.197–11.403, *p* = 0.023). Oncology patients: AdOR 2.587 (95% CI: 1.175–5.696, *p* = 0.018).

This consistent finding suggests that sociocultural, biological, or social factors contribute to the stronger inclination toward TAM among females [73,74,75]. The stronger association among healthcare professionals (AdOR 3.695) compared to oncology patients (AdOR 2.587) could indicate that healthcare workers are more familiar or open to these practices, even within the context of evidence-based medicine. Additionally, knowledge of traditional medicine was found to be a significant factor in influencing T&CM use (AdOR 1.689, 95% CI: 1.004–2.840, *p* = 0.048) [70]. However, in stratified analyses by group, this factor did not retain significance, suggesting that general awareness may play a less dominant role in influencing T&CM use compared to professional background or personal health needs [76].

The comparison between oncology patients and healthcare professionals reveals distinct patterns in the use of specific T&CM therapies: Herbal Products: Oncology patients reported significantly higher use (43.4%) compared to healthcare professionals (15.8%, *p* < 0.001), reflecting the common use of herbs for managing cancer symptoms or side effects. Homeopathy: More common among oncology patients (9.2%) than healthcare professionals (3.3%, *p* = 0.036), suggesting its appeal as a complementary approach to managing chronic illnesses like cancer. Consumption of exotic animals/venoms: Exclusive to oncology patients (4.8%, *p* = 0.004), underscoring the cultural and regional acceptance of this practice rooted in indigenous traditions. Non-Conventional Nutritional Interventions: Healthcare professionals reported slightly higher use (3.3%) than oncology patients (0.4%, *p* = 0.039), reflecting their awareness of emerging dietary trends. Combined Therapies: Oncology patients were more likely to use two or more therapies (13.2% vs. 6.6%, *p* = 0.042), suggesting that individuals facing complex health challenges are more inclined to integrate multiple alternative treatments alongside conventional ones.

The findings emphasize the importance of healthcare providers recognizing and understanding patients’ use of TAM, particularly in culturally diverse settings. Integrative approaches that respect traditional practices while ensuring evidence-based care could improve patient–provider relationships and enhance treatment adherence. Educational initiatives aimed at raising awareness among oncology patients about safe and effective T&CM practices could be valuable. Additionally, structuring policies that integrate T&CM into oncology care could create a more holistic framework for cancer management [76,77].

This study is limited by its cross-sectional design, which prevents causal conclusions. Self-reported data may also be subject to recall or social desirability bias. Future studies should focus on longitudinal analyses of T&CM use and evaluate the safety and efficacy of specific therapies in complementing conventional treatments. Expanding systematic reviews through platforms like the Cochrane Complementary Medicine Field could help evaluate the clinical evidence for T&CM, particularly in oncology. Future research should also explore the cultural, educational, and gender-specific factors influencing T&CM use. Longitudinal studies could investigate changes in T&CM usage over time and the influence of healthcare access and integrative medical practices. Additionally, examining how professional knowledge and gender intersect to shape healthcare behaviors could provide valuable insights for tailored patient care [5]. It is important to note that in the region of Mexico where this study was conducted, no healthcare institutions (including hospitals) provide traditional or complementary medicine treatments. Therefore, the treatments reported in this study were administered outside the conventional healthcare system. As a result, this report does not reflect the clinical experience of using such therapies within the analyzed hospital setting. However, the high frequency of their use highlights the need for these treatments to be integrated into or considered within healthcare frameworks. Additionally, no information was collected regarding whether the administration of these treatments was guided by an expert in alternative medicine or was a form of self-care. Future research is necessary to address this gap.

A second limitation of this study is the lack of detailed information in the form of use, dosage, and administration route of the alternative medicine products. Some patients and healthcare personnel were unable to provide these details, leading to the reporting of only the species used. In future studies, it would be valuable to obtain this additional information, though it is important to consider that, due to cultural sensitivity and the protection of ancestral knowledge, gathering these data may be challenging. A more delicate and respectful approach is recommended for collecting this information in future research. Finally, some perspectives for future studies will also explore the differences between traditional and complementary medicine (T&CM) users and non-users among oncology patients. While this approach presents a complex issue, especially when considering non-users, it could yield valuable insights into the factors that prevent certain patients from utilizing T&CM despite its cultural prevalence. According to studies from 2016, it is estimated that 90% of Mexicans continue to utilize botanical remedies, either solely or in conjunction with other forms of medicine, consuming 3500 tons of medicinal plants each month [78]. This fact highlights the deep-rooted tradition of T&CM use, which could be explored further, especially when analyzing non-users and the reasons behind their choices.

The final limitation of this study is that healthcare professionals, including doctors, nurses, and other staff members, were grouped into a single category. This prevents a more detailed analysis of the differences in how various disciplines, such as oncology, nursing, or social work, perceive and integrate T&CM. In future studies, it is recommended to expand the healthcare professional population in the study and compare its different roles, such as oncologists versus nursing staff, or social work versus other healthcare areas, which will provide a more nuanced understanding of how T&CM is perceived and integrated across various disciplines within the hospital setting. This would offer a dynamic view of how healthcare professionals interact with T&CM in different contexts, such as clinical care versus patient support roles. In the same role, comparative analysis with other hospitals within the same region (Western Mexico) or across different regions of the country could broaden the scope of this research. By examining regional differences in the use and acceptance of T&CM, the study could provide a more comprehensive understanding of how cultural and institutional factors influence the integration of T&CM in cancer care.

Future studies should explore the potential risks and benefits of integrating T&CM into oncology care, particularly in relation to its interaction with conventional cancer treatments. Given the widespread use of T&CM among oncology patients, it is critical to examine how these therapies may affect treatment adherence and the overall efficacy of standard cancer treatments. One area of particular concern is the potential for T&CM to reduce adherence to conventional treatment plans, as patients may prioritize alternative therapies over-prescribed medical regimens. Therefore, future research should investigate the possible risks of decreased adherence among oncology patients, as well as the broader implications of replacing conventional treatments with T&CM. Additionally, studies should consider variables such as dietary habits and interactions between T&CM and conventional treatments, as these factors may significantly influence treatment outcomes. By addressing these gaps in knowledge, future research can contribute to a more comprehensive understanding of how T&CM can be safely integrated into oncology care without compromising the effectiveness of established therapies.

## 5. Conclusions

In conclusion, this study provides important insights into the use of T&CM among oncology patients and healthcare personnel, highlighting the role of cultural, gender, and professional factors in shaping treatment preferences. By addressing these factors, future healthcare interventions can bridge the gap between traditional and conventional medicine, potentially improving patient outcomes. Integrating T&CM into standard care could enhance treatment effectiveness by considering patients’ cultural beliefs and preferences, leading to better adherence and overall satisfaction with their treatment regimen. However, it is essential to ensure that this integration is performed safely and collaboratively with conventional treatments to avoid potential risks and to optimize patient well-being.

## Figures and Tables

**Table 1:** General characteristics and use of traditional and alternative medicine among patients and healthcare professionals at an oncology hospital in Western Mexico.

Characteristic	All	Oncology Patients	Healthcare Personnel (*n* = 152)			*p*
			Physicians	Nurses	Other Staff	
	(*n* = 382)	(*n* = 230)	(*n* = 46)	(*n* = 54)	(*n* = 52)	
Age (years)	50.6 ± 14.1	56.0 ± 14.0	41.7 ± 8.0	40.4 ± 11.1	44.8 ± 9.1	<0.001
BMI (kg/m^2^)	28.7 ± 5.2	29.0 ± 5.5	27.2 ± 3.9	26.5 ± 5.3	28.7 ± 5.2	<0.001
Female (%)	74.1% (*n* = 283)	75.7% (*n* = 174)	52.2% (*n* = 24)	96.3% (*n* = 52)	63.5% (*n* = 33)	<0.001
Knows what traditional/alternative medicine is (%)	50.5% (*n* = 193)	39.6% (*n* = 91)	69.6% (*n* = 32)	66.7% (*n* = 36)	65.4% (*n* = 34)	<0.001
Has used traditional medicine (%)	87.7% (*n* = 335)	85.7% (*n* = 197)	87.0% (*n* = 40)	94.4% (*n* = 51)	90.4% (*n* = 47)	0.257
Frequent current use (%)	35.4% (*n* = 135)	46.3% (*n* = 106)	6.5% (*n* = 3)	33.3% (*n* = 18)	15.4% (*n* = 8)	<0.001
Severe/life-threatening illness (%)	65.2% (*n* = 249)	100.0% (*n* = 230)	17.4% (*n* = 8)	13.0% (*n* = 7)	7.7% (*n* = 4)	<0.001
Takes allopathic medication (%)	71.7% (*n* = 274)	100.0% (*n* = 230)	37.0% (*n* = 17)	31.5% (*n* = 15)	19.2% (*n* = 10)	<0.001
Homemaker (%)	31.2% (*n* = 119)	51.7% (*n* = 119)	0.0%	0.0%	0.0%	<0.001
High school education or above (%)	59.4% (*n* = 227)	34.3% (*n* = 79)	100.0% (*n* = 46)	100.0% (*n* = 54)	92.3% (*n* = 48)	<0.001
Lives with a partner (%)	63.6% (*n* = 243)	64.8% (*n* = 149)	56.5% (*n* = 26)	66.7% (*n* = 36)	61.5% (*n* = 32)	0.699
Low socioeconomic level (%)	31.8% (*n* = 141)	52.8% (*n* = 121)	0.0%	0.0%	0.0%	<0.001
Catholic (%)	84.8% (*n* = 324)	86.5% (*n* = 199)	82.6% (*n* = 38)	83.3% (*n* = 45)	80.8% (*n* = 42)	0.703
Diabetes (%)	12.8% (*n* = 49)	18.7% (*n* = 43)	6.5% (*n* = 3)	0.0%	5.8% (*n* = 3)	<0.001
Hypertension (%)	21.5% (*n* = 82)	29.3% (*n* = 67)	15.2% (*n* = 7)	7.4% (*n* = 4)	7.7% (*n* = 4)	<0.001

A total of 382 individuals were included in the study, consisting of 230 oncology patients and 152 healthcare professionals from the hospital where the study was conducted. Values are presented as mean ± standard deviation for continuous variables and as percentages (*n*) for categorical variables. “Frequent current use” refers to the use of traditional or alternative medicine two or more times per week. *p*-values were calculated using t-tests for continuous variables and chi-square tests for categorical variables.

**Table 2:** Cancer Diagnoses and Conventional Care among Oncology Patients.

Cancer Diagnosis	Oncology Patients	Conventional Care for Cancer	Oncology Patients
Breast	46.1% (*n* = 106)	Palliative care	11.7% (*n* = 27)
Cervical	11.3% (*n* = 26)	Chemotherapy	33.5% (*n* = 77)
Prostate	11.3% (*n* = 26)	Radiotherapy	11.7% (*n* = 27)
Colorectal	8.7% (*n* = 20)	Chemoradiotherapy	7.0% (*n* = 16)
Ovarian	6.1% (*n* = 14)	Patient in follow-up	27.8% (*n* = 64)
Non-Hodgkin lymphoma	2.6% (*n* = 6)	I haven’t received it yet	6.5% (*n* = 15)
Thyroid	2.6% (*n* = 6)	I don’t know	1.7% (*n* = 4)
Lung	2.2% (*n* = 5)		
Other cancers	9.1% (*n* = 21)		

Data represent the distribution of cancer diagnoses and the types of conventional cancer care received by oncology patients (*n* = 230) included in the study. Values are expressed as percentages (%) and absolute numbers (*n*).

**Table 3:** Comparison of different types of traditional or alternative medicine used by healthcare professionals and oncology patients.

Characteristic	All	Healthcare Personnel	Oncology Patients	*p*
	(*n* = 382)	(*n* = 152)	(*n* = 230)	
Herbal products (%)	63.87% (*n* = 244)	49.0% (*n* = 75)	73.6% (*n* = 169)	<0.001
Non-conventional nutritional interventions (%)	13.35% (*n* = 51)	32.5% (*n* = 49)	0.7% (*n* = 2)	<0.001
Homeopathy (%)	15.96% (*n* = 61)	18.5% (*n* = 28)	14.2% (*n* = 33)	0.350
Yoga (%)	7.85% (*n* = 30)	14.6% (*n* = 22)	3.4% (*n* = 8)	0.001
Acupuncture (%)	4.97% (*n* = 19)	7.3% (*n* = 11)	3.4% (*n* = 8)	0.198
Consumption of exotic animals/venoms (%)	4.9% (*n* = 19)	2.0% (*n* = 3)	7.1% (*n* = 16)	0.037
Bioelectromagnetic therapy (%)	2.0% (*n* = 8)	2.0% (*n* = 3)	2.0% (*n* = 5)	0.999
Spiritual Cleanse (%)	0.7% (*n* = 3)	0.0%	1.4% (*n* = 3)	0.244
Two or more therapies (%)	20.1% (*n* = 77)	18.5% (*n* = 28)	21.6% (*n* = 49)	0.564
Three or more therapies (%)	6.0% (*n* = 23)	6.6% (*n* = 10)	5.5% (*n* = 13)	0.809

Values are presented as percentages (*n*). Statistical significance was determined using chi-square tests. “Non-conventional nutritional interventions” refers to dietary approaches outside mainstream recommendations. Consumption of exotic animals/venoms: a branch of traditional medicine in many cultures, especially in indigenous and rural communities, involving the use of fats, bones, skins, venoms, and other animal derivatives. Bioelectromagnetic therapy uses electromagnetic fields to influence biological processes, aiming to relieve pain, reduce inflammation, and promote healing. Spiritual Cleanse: practice to clear negative energy from the body, mind, or spirit, often through rituals, prayers, herbs, chanting, or burning sacred substances like sage, aiming to restore balance, promote healing, and enhance emotional and spiritual well-being. The data for “Two or more therapies” and “Three or more therapies” were obtained by summing the number of therapies indicated by the patient during the interview.

**Table 4:** Multivariate logistic regression to detect factors associated with the use of traditional or alternative medicine.

Variable	Ad OR	95% CI		*p*
Factors associated with all participants				
Female	2.816	1.487	5.331	0.001
Knows Traditional Medicine Concept	1.689	1.004	2.840	0.048
Factors associated with healthcare personnel				
Female	3.695	1.197	11.403	0.023
Factors associated with oncology patients				
Female	2.587	1.175	5.696	0.018

A multivariate binary logistic regression analysis was conducted to determine adjusted odds ratios (AdORs) with 95% confidence intervals (CIs) and *p*-values. The backward stepwise selection method was employed to identify the most parsimonious model, with entry and removal probabilities set at 0.10 and 0.15, respectively (only the final step is presented). The model included variables that showed significant differences between patients with and without cancer (as outlined in Table 1), along with other relevant factors such as age, hormone use, being 50 years or older, female gender, cancer status (reference group: healthcare personnel), Catholic religion, high school education or higher, and obesity.

**Table 5:** Comparison of Frequently Used Traditional and Alternative Medicine Types Between Healthcare Professionals and Oncology Patients.

Characteristic	All	Healthcare Personnel	Oncology Patients	*p*
	(*n* = 382)	(*n* = 152)	(*n* = 230)	
Herbal products (%)	32.4% (*n* = 123)	15.8% (*n* = 23)	43.4% (*n* = 100)	<0.001
Non-conventional nutritional interventions (%)	1.6% (*n* = 6)	3.3% (*n* = 5)	0.4% (*n* = 1)	0.039
Homeopathy (%)	6.8% (*n* = 26)	3.3% (*n* = 5)	9.2% (*n* = 21)	0.036
Yoga (%)	2.4% (*n* = 9)	3.3% (*n* = 5)	1.7% (*n* = 4)	0.493
Acupuncture (%)	1.6% (*n* = 6)	1.3% (*n* = 2)	1.7% (*n* = 4)	0.999
Consumption of exotic animals/venoms (%)	2.9% (*n* = 11)	0.0%	4.8% (*n* = 11)	0.004
Bioelectromagnetic therapy (%)	1.3% (*n* = 5)	1.3% (*n* = 2)	1.3% (*n* = 3)	0.999
Spiritual Cleanse (%)	0.5% (*n* = 2)	0.0%	0.9% (*n* = 2)	0.519
Two or more therapies (%)	10.5% (*n* = 40)	6.6% (*n* = 10)	13.2% (*n* = 30)	0.042
Three or more therapies (%)	2.9% (*n* = 11)	2.6% (*n* = 4)	3.1% (*n* = 7)	0.999

“*n*” represents the number of participants in each group using the respective types of alternative medicine. For details on the alternative medicine types listed in this table, refer to the explanations in Table 3.

**Table 6:** Multivariate Logistic Regression to Detect Factors Associated with Current and Frequent Use of Alternative Medicine.

Variable	Ad OR	95% CI		*p*
Factors associated with all participants				
Oncology patient	6.573	3.732	11.574	<0.001
BMI ≥ 30 (obesity)	0.610	0.376	0.990	0.045
Knowledge of traditional medicine	2.690	1.639	4.415	<0.001
Factors associated with healthcare personnel				
Married or living with partner	0.208	0.074	0.583	0.003
Hypertension (HTA)	5.838	1.330	25.621	0.019
Age ≥ 50 years	4.267	1.496	12.171	0.007
BMI ≥ 30 (obesity)	0.170	0.036	0.798	0.025
Factors associated with oncology patients				
Knowledge of traditional medicine	2.650	1.533	4.581	<0.001

Multivariate logistic regression analysis identifying factors associated with the current and frequent use of alternative medicine in all participants, healthcare personnel, and oncology patients.

**Table 7:** Opinions on traditional and complementary medicine among healthcare professionals and oncology patients at an oncology hospital in Western Mexico.

Characteristic	All	Healthcare Professionals	Oncology Patients	*p*
	(*n* = 382)	(*n* = 152)	(*n* = 230)	
It works well for some diseases, but should be used alongside conventional medicine	64.9% (*n* = 248)	48.7% (*n* = 74)	75.7% (*n* = 174)	<0.001
It works well for some diseases, where it can be the sole treatment	14.4% (*n* = 55)	24.3% (*n* = 37)	7.8% (*n* = 18)	<0.001
It works well for all diseases, and can be the only treatment	5.5% (*n* = 21)	9.2% (*n* = 14)	3.0% (*n* = 7)	0.012
I respect its use, but would not use it	14.1% (*n* = 54)	15.8% (*n* = 24)	13.0% (*n* = 30)	0.457
I do not agree	1.0% (*n* = 4)	2.0% (*n* = 3)	0.4% (*n* = 1)	0.306
Positive opinion of its use	84.8% (*n* = 324)	82.2% (*n* = 125)	86.5% (*n* = 199)	0.308

Percentages represent the proportion of participants in each group who selected each opinion. *p*-values were calculated using a chi-square test to compare the responses between healthcare professionals and oncology patients.

**Table 8:** Comparison of Natural Product Usage Among Oncology Patients and Healthcare Personnel.

Category	Product	Scientific Name	Oncology Patients % (*n*)	Healthcare Personnel % (*n*)
Herbal products	Guanabana or Soursop	*Annona muricata*	33.7 (78)	NA
	Maguey	*Agave* spp.	9.3 (21)	NA
	Aloe Vera	*Aloe vera*	4.1 (9)	2.8 (4)
	Kalanchoe	*Kalanchoe pinnata*	3.5 (8)	NA
	Guava	*Psidium guajava*	2.9 (7)	1.9 (3)
	Turmeric	*Curcuma longa*	1.7 (4)	2.8 (4)
	Cat’s Claw	*Uncaria tomentosa*	1.7 (4)	NA
	Chicory (in Spanish: chico corrioso)	*Cichorium intybus*	1.7 (4)	NA
	Arnica	*Arnica montana*	1.7 (4)	2.8 (4)
	Chamomile	*Matricaria chamomilla*	1.2 (3)	6.5 (10)
	Skunk Plant	*Paullinia pinnata*	1.2 (3)	9.3 (14)
	Garlic	*Allium sativum*	1.2 (3)	1.9 (3)
	Moringa	*Moringa oleifera*	1.2 (3)	NA
	Cuachalalate	*Amphipterygium adstringens*	1.2 (3)	NA
	Horsetail	*Equisetum arvense*	1.2 (3)	NA
	Sapote (in Spanish: zapote)	*Casimiroa edulis*	1.2 (3)	NA
	Hedgehog (in Spanish: erizo)	*Kroenleinia grusonii*	1.2 (3)	NA
	Mezcal	*Agave* spp.	1.2 (3)	NA
	Coconut	*Cocos nucifera*	1.2 (3)	NA
	Spearmint	*Mentha spicata*	NA	4.7 (7)
	Cinnamon	*Cinnamomum verum*	NA	4.7 (7)
	Abango	*Pulmonaria officinalis*	NA	3.7 (6)
	Rue (in Spanish: ruda)	*Ruta graveolens* L.	NA	3.7 (6)
	Horse Chestnut	*Aesculus hippocastanum*	NA	3.7 (6)
	Passionflower	*Passiflora incarnata*	NA	2.8 (4)
	Ginger	*Zingiber officinale*	NA	2.8 (4)
	Noni	*Morinda citrifolia*	NA	2.8 (4))
	Snake Plant (in Spanish:espada de ángel)	*Dracaena trifasciata*	NA	2.8 (4)
	Black Nightshade Root	*Solanum nigrum* L.	NA	2.8 (4)
	Rosemary	*Salvia rosmarinus*	NA	1.9 (3)
	Linden	*Tilia americana* var. *mexicana*	NA	1.9 (3)
	Lemongrass	*Cymbopogon citratus*	NA	1.9 (3)
	Parota	*Enterolobium cyclocarpum (Jacq.) Griseb*	NA	1.9 (3)
	Palo santo	*Bursera graveolens*	NA	1.9
Consumption of exotic animals/venoms	Propolis	*Apis mellifera Propolis*	NA	2.8 (4)
	Snake Meat	*Crotalus* spp.	2.9 (7)	NA
	Immunocal	*Whey Protein Product*	1.7 (4)	NA
	Iguana Meat	*Iguana iguana*	1.2 (3)	NA
	Venoms	*Scorpion* and *Rattlesnake Venom* (*Centruroides tecomanus* and *Crotalus* spp.)	1.2 (3)	NA
Unspecified	Unnamed Products *		4.1 (9)	7.5 (11)

NA: Not mentioned. * Patient/Staff did not know the name. Herbal products: includes herbal and natural products derived from plants. Consumption of exotic animals/venoms: a branch of traditional medicine in many cultures, especially in indigenous and rural communities, involving the use of fats, bones, skins, venoms, and other animal derivatives. The percentages reflect the consumption of natural products reported by oncology patients (*n* = 230) and healthcare professionals (*n* = 152). Products less commonly consumed by patients include lemon, rosemary, cinnamon, arnica, aloe vera, noni, neem, snake plant, nettle, cancerina, buzzard plant, cactus, muicle, spirulina, chia, sabal, “bad mother” plant, moronel, caiman, chlorine dioxide, ozone, plantain, cow’s tongue plant, shark cartilage, and corn silk. Products less commonly consumed by healthcare professionals include bay leaf, mastranzo, nettle, mullein, stevia, orange blossom, basil, milk thistle, cuachalalate, horsetail, dandelion, stone flower, epazote, marijuana or derivatives, and homeopathy.

## Data Availability

The original contributions presented in the study are included in the article/Supplementary Materials; further inquiries can be directed to the corresponding author/s.

## Supplementary Materials

The following supporting information can be downloaded at: https://www.mdpi.com/article/10.3390/curroncol32020071/s1, Section S1: Glossary of Employed Terms. Section S2: Developed Questionnaire (Spanish and English Translation).
