# Supplementary material for: Comparing Perspectives on Traditional and Complementary Medicine Use in Oncology: Insights from Healthcare Professionals and Oncology Patients in Western Mexico
Source: Curr Oncol. 2025 Jan 28;32(2):71. doi: 10.3390/curroncol32020071 (PMC11854627; doi:10.3390/curroncol32020071)
Supplement: Supplementary file 1 [file curroncol-32-00071-s001.zip › curroncol-3389256-supplementary.pdf]

Comparing Perspectives on Traditional and Complementary Medicine Use in Oncology: Insights from Healthcare Professionals and Oncology Patients in Western Mexico

Section S1: Glossary of Employed Terms

- Herbal/Plants
  Refers to the use of plants and herbs for promoting health and treating illnesses. Herbs can be consumed in various forms such as teas, extracts, or supplements. Many of these practices are rooted in ancient traditions, although some have been supported by scientific studies (30).
- Consumption of Exotic Animals or Their Products
  Involves the use of products derived from rare or exotic animals, such as body parts (bones, skin, etc.), for medicinal or therapeutic purposes. These practices may be linked to cultural or traditional beliefs, though they often lack scientific backing (31,41)
- Acupuncture
  Acupuncture is a therapeutic technique from traditional Chinese medicine that involves inserting fine needles into specific points on the body to relieve pain and treat various health conditions. It is believed to improve the flow of energy (Qi) through the body (40).
- Yoga
  Yoga is a discipline that combines physical exercise, breath control, and meditation. It is used to promote flexibility, reduce stress, and improve mental and physical well-being. Though originating in India, yoga is practiced worldwide as a method of relaxation and self-care (39).
- Homeopathy
  Homeopathy is a therapeutic approach that uses diluted substances in water or alcohol to treat various conditions. Homeopaths believe that these substances, even when extremely diluted, can stimulate the body to heal itself (37).
- Non-conventional Nutritional Therapies
  Refers to alternative approaches to nutrition that go beyond conventional diets, including the use of supplements, radical dietary changes, or specific foods as therapy to treat illnesses or improve overall health (33).
- Zoo Therapy (Use of Animals)

This therapeutic approach involves interaction with animals (e.g., dogs, horses, etc.) to improve emotional and physical well-being. It is particularly used in rehabilitation settings or for treating mental health conditions (36).

- Bioelectromagnetic Therapy
  This therapy uses low-frequency electromagnetic fields to treat various conditions. Devices that apply this therapy are used to alleviate pain and speed recovery from certain disorders, although scientific evidence regarding its effectiveness is limited (38,35).
- Spiritual Cleansing
  A set of practices aimed at removing or purifying negative spiritual or energetic influences from a person's body or environment. These practices may include rituals, prayers, or the use of herbs or specific items, based on traditional or spiritual beliefs (32,34).

30. World Health Organization (WHO) *WHO Monographs on Selected Medicinal Plants*; World Health Organization, Ed.; 1st ed.; WHO Library Cataloguing in Publication Data: Geneva, Switzerland, 1999; Volume 1.
31. Warwick, C.; Steedman, C. Wildlife-Pet Markets in a One-Health Context. *Int. J. One Health* **2021**, *7*, 42–64. https://doi.org/10.14202/IJOH.2021.42-64.
32. Warren, D. The Interpretation of Change in a Ghanaian Ethnomedical Study. *Hum. Organ.* **1978**, *37*, 73–77. https://doi.org/10.17730/humo.37.1.f24121j323r7507h.
33. U.S. Food and Drug Administration Dietary Supplements: What You Need to Know. Available online: https://ods.od.nih.gov/factsheets/WYNTK-Consumer/ (accessed on 10 January 2025).
34. Mata-Pinzón, S. Plantas Medicinales Para El Tratamiento Del Susto y Mal de Ojo. Análisis de Sus Posibles Efectos Sobre El Sistema Nervioso Central Por Vía Transdérmica e Inhalatoria. *Etnobiología* **2018**, *16*, 30–47.
35. Markov, M.S. Pulsed Electromagnetic Field Therapy History, State of the Art and Future. *Environmentalist* **2007**, *27*, 465–475. https://doi.org/10.1007/s10669-007-9128-2.
36. Fine, A.H. Handbook on Animal-Assisted Therapy. Theoretical Foundations and Guidelines for Practice; Ed.; 3rd ed.; Elsevier: Pomona, CA, USA, 2010; Volume 1, ISBN 9780123814531.
37. Ernst, E. A Systematic Review of Systematic Reviews of Homeopathy. *Br. J. Clin. Pharmacol.* **2002**, *54*, 577–582. https://doi.org/10.1046/j.1365-2125.2002.01699.x.
38. Chang, H.-Y.; Huang, Y.-Y.; Chung, C.-J.; Liu, F.-H. A Comparative Analysis of Complementary Therapies Use among Patients Attending Diabetic Clinics in Taiwan: 2007 vs. 2023. *BMC Complement. Med. Ther.* **2023**, *23*, 455. https://doi.org/10.1186/s12906-023-04299-9.
39. Cameron, M.E.; Cheung, C.K. Yoga. In *Complementary Therapies in Nursing*; Lindquist, R., Mary Fran, T., Snyder, M., Eds.; Springer Publishing Company: New York, NY, USA, 2022; Volume 1, pp. 162–174.
40. Birch, S.; Lee, M.S.; Kim, T.-H.; Alraek, T. Historical Perspectives on Using Sham Acupuncture in Acupuncture Clinical Trials. *Integr. Med. Res.* **2022**, *11*, 100725. https://doi.org/10.1016/j.imr.2021.100725.
41. Ayala Enriquez, M.I.; Garcia Flores, A.; Montes De Oca, E.R.; Pino Moreno, J.M.; García Lara, F. Uso y Manejo Tradicional de La Fauna Silvestre Por Nahuas de Santa Catarina, Tepoztlán, Morelos, México. *Caldasia* **2022**, *45*, 36–48. https://doi.org/10.15446/caldasia.v45n1.92451.

Section S2: Developed Questionnaire (Spanish and English Translation)

This section presents the developed questionnaire, both in Spanish and its English translation. The questionnaire includes 31 questions divided into four sections, each designed to assess different aspects related to traditional and complementary (alternative) medicine. The Spanish version is followed by its corresponding English translation for clarity and consistency.

Spanish version

Instrucciones: Previa explicación y firma de consentimiento informado, este instrumento debe aplicarse en forma de entrevista, donde un encuestador hace las preguntas y captura la información según responda el participante

Folio de participante número_______________

SECCIÓN 1: CARACTERÍSTICAS UNIVERSALES, SOCIODEMOGRÁFICAS Y CLÍNICAS.

1. **Edad**: ______años
2. **Peso Kg**__________ **Estatura (metros)**_________
3. **Localidad y Estado donde vive**___________ **y donde nació**_____________
4. **Se identifica con algún grupo indígena**___________________
5. **Género**:
    a. ☐ Masculino
    b. ☐ Femenino
    c. ☐ Otro (especificar): _________
6. **Nivel educativo**:
    a. ☐ Sin educación formal
    b. ☐ Primaria
    c. ☐ Secundaria
    d. ☐ Preparatoria
    e. ☐ Licenciatura

f. ☐ Posgrado

7. **Estado civil**:

    a. ☐ Soltero

    b. ☐ Casado

    c. ☐ Divorciado

    d. ☐ Viudo

8. **Mencione las enfermedades que padece**

    ____

    ____

    ____

9. **Que medicamentos o tratamientos convencionales utiliza actualmente**

    a. ☐ Ninguno
    b. ☐Los siguientes:

    ____

    ____

    ____

10. **Nivel socioeconómico (ver criterios según (acorde a los criterios de: Mexican Association of Market Intelligence and Opinion Agencies (AMAI)**:

    a. ☐ Bajo (D y E)

    b. ☐ Medio (C)

    c. ☐ Alto (A y B)

11. **¿Cuál es su ocupación o trabajo?**

    a. ☐ Ama de casa

    b. ☐ Desempleado

    c. ☐ Empleado o con ingresos propios

    d. ☐ Jubilado

12. **En caso de trabajar actualmente, ¿cuál es su profesión o labor?**

    a. ☐ Trabaja en el sector salud, Profesión o labor: ______________________

b. ☐ No trabaja en el sector salud, Profesión o labor: ___________________

c. ☐ No trabaja

13. ¿Alguna vez se ha tenido una enfermedad que ha puesto en peligro su vida?

a. ☐ Si

b. ☐ No

14. **¿Cuál es su diagnóstico principal (si es paciente oncológico)?**

a. ☐ Cáncer de mama
b. ☐ Cáncer cervical
c. ☐ Cáncer de próstata
d. ☐ Cáncer colorrectal
e. ☐ Cáncer de ovario
f. ☐ Linfoma no-Hodgkin
g. ☐ Otro (especificar): ___________

SECCIÓN 2: CONOCIMIENTO SOBRE MEDICINA TRADICIONAL Y ALTERNATIVA

15. **¿Conoce qué es la medicina tradicional o alternativa?**

a. ☐ Sí

b. ☐ No

16. Defina como usted considere, el concepto de medicina tradicional y alternativa:

____
____
____

17. **¿Cuál de las siguientes opciones describe mejor su conocimiento de la medicina tradicional o alternativa?**

a. ☐ Entiendo que incluye prácticas como hierbas, acupuntura, yoga, etc.

b. ☐ Solo sé que son tratamientos diferentes a la medicina convencional.

c. ☐ No tengo claro lo que es.

SECCIÓN 3: USO DE TRATAMIENTOS TRADICIONALES Y ALTERNATIVOS

Instrucciones: de la pregunta 17 a la 21, se refiere al uso de medicina tradicional y alternativa alguna vez en la vida.

18. **¿Ha utilizado alguna vez tratamientos tradicionales o alternativos?**
    a. ☐ Sí
    b. ☐ No

19. **¿Para cuales enfermedades utilizó un tratamiento tradicional o alternativo?**

    ____

    ____

20. **Mencione que tipo de medicinal o alternativa que ha utilizado**
    a. ☐ Plantas/herbales
    b. ☐ Consumo de animales exóticos o sus productos (especificar)______________________________
    c. ☐ Acupuntura
    d. ☐ Yoga
    e. ☐ Homeopatía
    f. ☐ Terapias no convencionales de nutrición
    g. ☐ Terapia zooterapia (uso de animales)
    h. ☐ Bioelectromagnética
    i. ☐ Limpieza espiritual
    j. ☐ Otro (especificar): __________

21. **Mencione el nombre de los productos que utilizo y como los utilizó (mencione partes usadas de plantas-hojas, flores, raíces, cortezas, etc., y animales):**

    ____

    ____

    ____

    ____

22. **¿Con qué frecuencia utilizó estos tratamientos?**

a. ☐ Menos de una vez al mes

b. ☐ Una vez al mes

c. ☐ Dos o más veces a la semana

Instrucciones: de la pregunta 22 a la 26, se refiere al uso de medicina tradicional y alternativa desde el diagnostico de su padecimiento actual, siendo este el motivo por el que acude a este centro de salud. En caso de no ser paciente de este centro médico, se refiere a tratamientos en el periodo actual.

23. **Actualmente ¿Utiliza tratamientos tradicionales o alternativos?**

a. ☐ Sí

b. ☐ No

24. ¿Para cuales enfermedades utiliza actualmente un tratamiento tradicional o alternativo?

____

____

25. Mencione que tipo de medicinal o alternativa utiliza actualmente. Puede señalar mas de una respuesta.

a. ☐ Plantas/herbales
b. ☐ Consumo de animales exóticos o sus productos (especificar)________________________
c. ☐ Acupuntura
d. ☐ Yoga
e. ☐ Homeopatía
f. ☐ Terapias no convencionales de nutrición
g. ☐ Terapia zoo terapia (uso de animales)
h. ☐ Bioelectromagnética
i. ☐ Limpieza espiritual
j. ☐ Otro (especificar): _________

26. Mencione el nombre de los productos que utiliza actualmente, y como lo utiliza:

____

____

____

27. **¿Con qué frecuencia utiliza actualmente estos tratamientos?**

a. ☐ Menos de una vez al mes

b. ☐ Una vez al mes

c. ☐ Dos o más veces a la semana

28. Mencione los efectos adversos o contraproducentes que le han ocasionado los tratamientos de la Medicina Tradicional y Alternativa

a. ☐ No he presentado efectos adversos

b. ☐ Si he presentado lo siguiente: ____________________________________

____

29. ¿Quién le recomendó el uso de medicina tradicional o alternativa? Señale la opción que mejor represente el motivo por el cual decidió usar dicha medicina.

a. ☐ Familiar

b. ☐ Amigo o conocido

c. ☐ Personal de salud

d. ☐ TV

e. ☐ Revistas

f. ☐ Radio

g. ☐ Medio por Internet

h. ☐ Otro, menciónelo: _________________________________

SECCIÓN 4: OPINIÓN SOBRE LA MEDICINA TRADICIONAL Y ALTERNATIVA

30. **¿Qué opina sobre la medicina tradicional y alternativa?**

a. ☐ Funciona bien para algunas enfermedades y debe usarse junto con la medicina convencional.

b. ☐ Funciona bien para algunas enfermedades y puede ser el tratamiento único.

c. ☐ Funciona bien para todas las enfermedades y puede ser el único tratamiento.

d. ☐ No creo que sea efectivo en ninguna enfermedad.

31. **¿Cree que la medicina tradicional o alternativa puede interferir con los tratamientos convencionales?**

a. ☐ Sí

b. ☐ No

c. ☐ No estoy seguro/a

English version

Instructions: After providing an explanation and obtaining signed informed consent, this instrument should be administered as an interview, where the interviewer asks the questions and records the participant's responses.
Participant ID Number: ________________

SECTION 1: UNIVERSAL, SOCIODEMOGRAPHIC, AND CLINICAL CHARACTERISTICS

1. **Age:** _______ years
2. **Weight (kg):** ___________ **Height (meters):** ___________
3. **Locality and state where you live:** _____________ **Place of birth:** _____________
4. **Do you identify with an indigenous group?** _____________
5. **Gender:**
   a. ☐ Male
   b. ☐ Female
   c. ☐ Other (specify): _____________
6. **Education level:**
   a. ☐ No formal education
   b. ☐ Primary school
   c. ☐ Secondary school
   d. ☐ High school
   e. ☐ Bachelor's degree
   f. ☐ Postgraduate degree

7. **Marital status:**
   a. ☐ Single
   b. ☐ Married
   c. ☐ Divorced
   d. ☐ Widowed
8. **List the diseases you currently have:**
   ____
   ____
   ____
9. **What medications or conventional treatments are you currently using?**
   a. ☐ None
   b. The following:
   ____
   ____
10. **Socioeconomic level** (according to the criteria of the **Mexican Association of Market Intelligence and Opinion Agencies (AMAI):**
   a. ☐ Low (D and E)
   b. ☐ Middle (C)
   c. ☐ High (A and B)
11. **What is your occupation or work?**
   a. ☐ Homemaker
   b. ☐ Unemployed
   c. ☐ Employed or self-employed
   d. ☐ Retired
12. **If currently employed, what is your profession or job?**
   a. ☐ Works in the health sector. Profession or role: ______________________
   b. ☐ Does not work in the health sector. Profession or role: ______________________
   c. ☐ Does not work
13. **Have you ever had an illness that put your life at risk?**
    1. ☐ Yes
    2. ☐ No
14. **What is your primary diagnosis (if an oncology patient)?**
   a. ☐ Breast cancer

b. ☐ Cervical cancer
c. ☐ Prostate cancer
d. ☐ Colorectal cancer
e. ☐ Ovarian cancer
f. ☐ Non-Hodgkin lymphoma
g. ☐ Other (specify): _____________

SECTION 2: KNOWLEDGE ABOUT TRADITIONAL AND ALTERNATIVE MEDICINE

14. **Do you know what traditional or alternative medicine is?**
a. ☐ Yes
b. ☐ No

15. **Define the concept of traditional and alternative medicine as you understand it:**

____
____
____

16. **Which of the following best describes your knowledge of traditional or alternative medicine?**
a. ☐ I understand it includes practices like herbs, acupuncture, yoga, etc.
b. ☐ I only know it involves treatments different from conventional medicine.
c. ☐ I'm not clear on what it is.

SECTION 3: USE OF TRADITIONAL AND ALTERNATIVE TREATMENTS

Instructions: For questions 17 to 21, refer to the use of traditional and alternative medicine at some point in your life.

17. **Have you ever used traditional or alternative treatments?**
a. ☐ Yes
b. ☐ No

18. **For which diseases did you use traditional or alternative treatments?**

____
____

19. **What type of traditional or alternative medicine did you use?**
   a. ☐ Herbal/plants
   b. ☐ Consumption of exotic animals or their products (specify): ____________________________
   c. ☐ Acupuncture
   d. ☐ Yoga
   e. ☐ Homeopathy
   f. ☐ Non-conventional nutritional therapies
   g. ☐ Zoo therapy (use of animals)
   h. ☐ Bioelectromagnetic therapy
   i. ☐ Spiritual cleansing
   j. ☐ Other (specify): __________

20. **Name the products you used and how you used them:**

   ____

   ____

21. **How often did you use these treatments?**
   a. ☐ Less than once a month
   b. ☐ Once a month
   c. ☐ Two or more times a week

Instructions: For questions 22 to 26, refer to the use of traditional and alternative medicine since the diagnosis of your current condition, which is the reason for visiting this health center. If the participant is not a patient here, refer to current treatments.

22. **Are you currently using traditional or alternative treatments?**
a. ☐ Yes
b. ☐ No

23. **For which diseases are you currently using traditional or alternative treatments?**
a. ____

24. **What type of traditional or alternative medicine are you currently using? (More than one answer can be indicated)**
a. ☐ Herbal/plants
b. ☐ Consumption of exotic animals or their products (specify): ____________________________
c. ☐ Acupuncture
d. ☐ Yoga

e. ☐ Homeopathy
f. ☐ Non-conventional nutritional therapies
g. ☐ Zoo therapy (use of animals,)
h. ☐ Bioelectromagnetic therapy
i. ☐ Spiritual cleansing
j. ☐ Other (specify): __________

25. **Name the products you are currently using and how you use them (mention the parts used from plants—leaves, flowers, roots, bark, etc.—and animals):**

____

____

26. **How often do you currently use these treatments?**
    a. ☐ Less than once a month
    b. ☐ Once a month
    c. ☐ Two or more times a week

27. **List any adverse or counterproductive effects you have experienced from traditional or alternative treatments:**
    a. ☐ I haven't experienced adverse effects.
    b. ☐ I have experienced the following:

    ____

    ____

28. **Who recommended the use of traditional or alternative medicine? Indicate the option that best represents the reason why you decided to use this medicine.**
    a. ☐ Family member
    b. ☐ Friend or acquaintance
    c. ☐ Health professional
    d. ☐ TV
    e. ☐ Magazines
    f. ☐ Radio
    g. ☐ Internet
    h. ☐ Other (specify): _____________

SECTION 4: OPINION ABOUT TRADITIONAL AND ALTERNATIVE MEDICINE

29. **What is your opinion about traditional and alternative medicine?**
    a. ☐ It works well for some diseases and should be used alongside

conventional medicine.

b. ☐ It works well for some diseases and can be the sole treatment.

c. ☐ It works well for all diseases and can be the only treatment.

d. ☐ I don’t believe it is effective for any disease.

30. **Do you think traditional or alternative medicine can interfere with conventional treatments?**

a. ☐ Yes

b. ☐ No

c. ☐ Not sure
